# COVID‐19 partial school closures and mental health problems: A cross‐sectional survey of 11,000 adolescents to determine those most at risk

**DOI:** 10.1002/jcv2.12021

**Published:** 2021-07-20

**Authors:** Karen L. Mansfield, Danielle Newby, Emma Soneson, Nemanja Vaci, Christoph Jindra, Galit Geulayov, John Gallacher, Mina Fazel

**Affiliations:** ^1^ Department of Psychiatry University of Oxford Oxford UK; ^2^ Department of Psychiatry University of Cambridge Cambridge UK; ^3^ Department of Psychology University of Sheffield Sheffield UK; ^4^ Institut zur Qualitätsentwicklung im Bildungswesen Humboldt‐Universität zu Berlin Berlin Germany

**Keywords:** adolescent, COVID‐19, mental health, school

## Abstract

**Background:**

Understanding adolescents' mental health during lockdown and identifying those most at risk is an urgent public health challenge. This study surveyed school pupils across Southern England during the first COVID‐19 school lockdown to investigate situational factors associated with mental health difficulties and how they relate to pupils' access to in‐school educational provision.

**Methods:**

A total of 11,765 pupils in years 8–13 completed a survey in June–July 2020, including questions on mental health, risk indicators and access to school provision. Pupils at home were compared to those accessing in‐school provision on risk and contextual factors and mental health outcomes. Multilevel logistic regression analyses compared the effect of eight risk and contextual factors, including access to in‐school provision, on depression, anxiety and self‐reported deterioration in mental wellbeing.

**Results:**

Females, pupils who had experienced food poverty and those who had previously accessed mental health support were at greatest risk of depression, anxiety and a deterioration in wellbeing. Pupils whose parents were going out to work and those preparing for national examinations in the subsequent school year were also at increased risk. Pupils accessing in‐school provision had poorer mental health, but this was accounted for by the background risk and contextual factors assessed, in line with the allocation of in‐school places to more vulnerable pupils.

**Conclusions:**

Although the strongest associations with poor mental health during school closures were established risk factors, further contextual factors of particular relevance during lockdown had negative impacts on wellbeing. Identifying those pupils at greatest risk for poor outcomes is critical for ensuring that appropriate educational and social support can be given to pupils either at home or in‐school during subsequent lockdowns.

## Graphical abstract

This study assessed mental health and change in wellbeing in a large, diverse sample of pupils during the first UK COVID‐19 school closures, comparing pupils who remained at home with those who were accessing in‐school provision, and adjusting for situational factors. Pupils accessing in‐school provision had poorer mental health, but this was accounted for by pre‐existing vulnerability and contextual factors. Pupils most likely to report deteriorations in wellbeing were female, reported socio‐economic deprivation, had previous mental health needs or upcoming examinations.

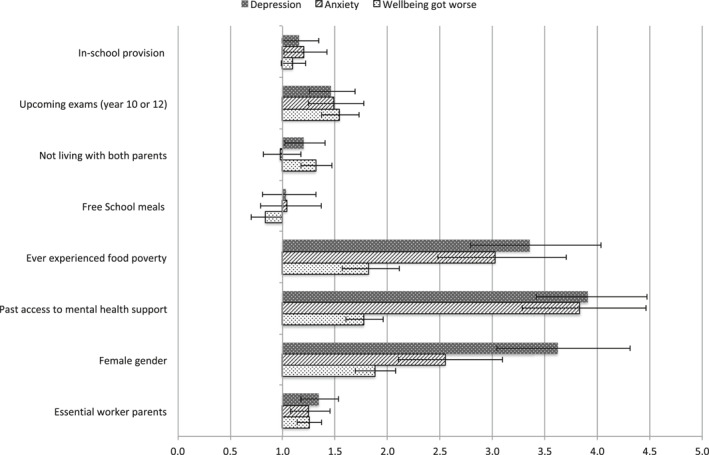

## INTRODUCTION

The impact of the COVID‐19 pandemic and resulting lockdowns on the mental health of young people is a significant societal concern (Courtney et al., 2020; Holmes et al., 2020; Lee, 2020; O'Connor et al., 2020; Townsend, 2020). Young people might be affected by the immediate consequences of full or partial school closures and changes to daily routine, potentially leading to reduced social contact, loneliness and negative impacts on their wellbeing (Brooks et al., 2020; Courtney et al., 2020; Orben et al., 2020). These detrimental effects are especially likely for school pupils who are already at risk of mental health difficulties.

**Key Points**

- Studies reporting the effects of school lockdown on adolescents' wellbeing are limited and results depend on the study sample
- This study assessed depression, anxiety, self‐reported change in wellbeing and situational risk in a large, diverse sample of pupils during the first UK COVID‐19 school closures. This is the first study to compare the wellbeing of pupils who remained at home with those who were accessing in‐school provision, adjusting for background factors
- Pupils accessing in‐school provision had poorer mental health, accounted for by background contextual factors. Pupils most likely to report deteriorations in wellbeing were female, reported socio‐economic deprivation and had previous mental health support or upcoming examinations
- The risk groups identified would benefit from a broad curriculum of support for education and wellbeing

In March 2020, the first UK COVID‐19 national lockdown commenced and schools were closed except to children whose parents were essential workers (also known as ‘critical’ or key workers), or those who were considered ‘vulnerable’, such as those with mental health needs or living in care (Cabinet Office, 2020; Department for Education, 2020). All other pupils were provided with varying degrees of educational support while at home. From 1 June, pupils in some year groups were also invited back to school, such as those approaching key national examinations. This implies that those offered places in school could be more at risk of mental health difficulties than those who remained at home for one or more reasons; either because they met the criteria for ‘vulnerability’, because their parents were performing essential roles outside the family home or because they needed to prepare for national examinations in the forthcoming academic year.

There was a concern that partial school closures may reinforce or exacerbate pre‐existing inequalities by disproportionally affecting young people who are already at increased risk of poor mental health (Armitage & Nellums, 2020; Van Lancker & Parolin, 2020; Viner et al., 2020). School closures might have some unique benefits to those pupils who frequently experience behavioural difficulties or peer victimisation at school, but it might be detrimental for many others with mental health problems due to loss of support (Courtney et al., 2020; Golberstein et al., 2020; Lee, 2020; YoungMinds, 2020). Cumulative risk factors within homes, such as domestic abuse, limited physical space, economic challenges and single parenthood, may contribute to increased adversity, potentially leading to both immediate and long‐term consequences for mental health (Clemens et al., 2020; Cluver et al., 2020; Courtney et al., 2020; Crawley et al., 2020; Gilbert et al., 2009; Usher et al., 2020).

To inform practice and policy for future periods of school lockdown and long‐term consequences, this study investigates which young people were most at risk of negative impacts during school closures on their mental health and wellbeing (Golberstein et al., 2020; Holmes et al., 2020; Lee, 2020; O'Connor et al., 2020) and how increased risk relates to whether or not pupils were getting access to in‐school educational provision. We used a large cross‐sectional survey of school pupils during June–July 2020 and compared established factors and lockdown‐specific demographic and situational factors on three different outcomes: clinical depression, clinical anxiety and pupils' self‐reported deterioration of their mental wellbeing.

## METHODS

### Design

OxWell is an annual cross‐sectional survey of schools and further education colleges (FECs) in Southern England (Mansfield & Fazel, 2020). This study analyses responses from pupils in years 8–13 (age 12 years and over), for whom the survey included the 25‐item Revised Children's Anxiety and Depression Scales (RCADS) (Ebesutani et al., 2012) and multiple questions to assess the risk of mental health disorders.

### Population

School pupils in years 8–13 (age 12–25 years) at state‐maintained and independent secondary schools and FECs (excluding special schools) in Oxfordshire, Berkshire, Buckinghamshire, Gloucestershire and Wiltshire, plus six schools in North Somerset and Bristol.

### Recruitment

Head teachers were invited to sign up their school via an email from their local authority in May–July 2020. All participating schools sent study information and opt‐out instructions to parents/guardians before providing information and login instructions directly to pupils or in some cases via parents.

### Ethical considerations

All participants included in these analyses gave active online assent to participate. Ethical approval (Ref. R62366/RE0010) was obtained from the University of Oxford Medical Sciences Division Research Ethics Committee.

### Measures

The OxWell survey includes over 200 core questions that are asked in annual surveys and additional questions that vary according to emerging hypotheses, described further in the study protocol (Mansfield et al., Submitted). All measures and questions selected for this study are detailed in online Tables S1 and S2. We selected mental health outcomes of depression and anxiety using RCADS (Chorpita et al., 2000; Ebesutani et al., 2012) and of the impact of school closures on mental wellbeing using a single item measure of pupils' perceived change to their wellbeing during lockdown. Predictors were selected that were background factors that could not have been influenced by the outcomes and were a proxy for established risk factors (relating to deprivation and vulnerability), or other characteristics relevant to the allocation of school places (essential worker parents and having upcoming exams). Accordingly, the indicators of socio‐economic deprivation selected were access to free school meals and experience of food poverty. The indicators of potentially increased vulnerability were female gender; previous access to mental health support (including within school support); and living circumstances (with both parents in one house compared to other configurations). Measures relevant to the situational risk factors especially relevant to lockdown were how often parents were going out to work (a proxy for essential workers) or being in school years 10 or 12 (approaching UK national examinations in 2021 and thus invited back to school from June 2020). ‘School’ and ‘school year’ were selected as control variables in order to adjust for the variance due to these factors.

### Analysis plan

For respondents who answered all 25 RCADS items, *t*‐scores for depression, anxiety and combined depression and anxiety were calculated (Chorpita et al., 2000). All *p*‐values were corrected for multiple testing using the false discovery rate, a recommended correction to minimise false positive rates for exploratory analyses in health studies (Glickman et al., 2014), and interpreted at the 95% confidence level.

#### Outcomes

To investigate which groups were most at risk of clinical depression and anxiety, we created binary RCADS outcomes using the diagnostic thresholds (*t*‐scores ≥ 70) (Chorpita, 2020). To investigate which groups were most likely to perceive their wellbeing to have deteriorated during lockdown, we created a binary outcome defining participants who reported their wellbeing to be ‘slightly worse’ or ‘much worse’ during lockdown.

#### Predictors

All predictors assessed were modelled as binary indicators. Predictor variables included demographic measures (female gender), socioeconomic indicators (free school meals, experience of food poverty), contextual indicators (previous access to mental health support, not living with both parents) and situational factors relevant to lockdown (upcoming examinations, parents in essential roles). The binary variable for year groups with upcoming examinations and invited back to school from June (years 10 and 12) used the remainder of pupils (years 8, 9, 11 and 13) as the reference group. Children of essential workers were identified by how many days their parents left the house to go to work (‘most days’ or ‘every day’), referenced against those whose parents only went out to work ‘sometimes’, ‘once or twice’ or ‘never’. A further binary variable compared pupils who were ‘in‐school’ during lockdown (those who had left the house to go to school at least once or twice) versus those who were ‘at home’ (pupils who had ‘never’ attended school during lockdown at the time of participation). This allowed a comparison of odds ratios (ORs) for those eligible (and taking up in‐school places) to the other individual situational risk and contextual indicators.

#### Models

Multilevel logistic regression analysis (R; lme4 package) (Bates et al., 2014; R Core Team, 2020) was used to calculate ORs for each factor for the three outcomes. In order to adjust for the variance across schools and year groups, accounting for the nested structure of the data, school and year group were included as random intercepts. The set of predictors was identical for each outcome (model), such that the regression coefficients reflect the independent contribution of each variable on the outcome, adjusted for all other variables. As a robustness test, we ran a very preliminary sensitivity analysis aiming to account for non‐response bias, which included weights based on ranking and auxiliary information for a subset of demographics that could be matched with the Office for National Statistics census data. We have reported results from the sensitivity analysis when this affected the interpretation of significance levels.

## RESULTS

### Participants

Of the 65,082 potentially eligible pupils in years 8–13 at the 84 secondary schools and 7 FECs, 14,352 accessed the survey. Pupils who did not access the survey were either not contacted by their school (some schools chose not to invite all relevant year groups), opted out by their parents (schools kept these records), did not read the survey information sent by their school (either due to lack of engagement or limited access to digital media) or chose not to participate. Participants were excluded from the analysis if they spent less than 10 min completing the survey or gave unrealistic or inconsistent responses, leaving 11,765 eligible participants (82% of those who accessed the survey). Of these, 10,095 answered all 25 RCADS questions, 10,633 reported perceived change to mental wellbeing and 10,784 provided information on how often they physically attended school, which defined the study population (see Table 1). A further 1252 participants had missing data for one or more of the remaining predictors and were not included in the logistic regression models. Sixty‐three percentage of the sample was female and response rates declined for older year groups. Participants were aged 12–21, and 315 (3% of the study population) were over 18 and technically adults. The prevalence in this sample of above the clinical threshold depression was 14% and for anxiety it was 10%, with 38% reporting their wellbeing to be worse during lockdown.

**TABLE 1:** Sample characteristics according to home or in‐school educational provision

Variables		Educational provision: *n* (%)			
		At home	In‐school	Total	*n* in comparison
		*N* = 7876	*N* = 2908	*N* = 10784	
Demographics (years grouped as binary indicator in models)	Gender (female)	4943 (63%)	1836 (64%)	6779 (63%)	10,709
	School year 8	2399 (31%)	520 (18%)	2919 (27%)	2919
	School year 9	2098 (27%)	562 (19%)	2660 (25%)	2660
	School year 10 (upcoming exams)	1324 (17%)	1136 (39%)	2460 (23%)	2460
	School year 11	794 (10%)	85 (3%)	879 (8%)	879
	School year 12 (upcoming exams)	957 (12%)	568 (20%)	1525 (14%)	1525
	School year 13	304 (4%)	37 (1%)	341 (3%)	341
Situational risk and contextual factors	Not living with both parents	1465 (19%)	652 (23%)	2117 (20%)	10,607
	Free school meals	584 (7%)	233 (8%)	817 (8%)	10,784
	Ever experienced food poverty	647 (9%)	312 (11%)	959 (9%)	10,390
	Past access to mental health support	1821 (23%)	835 (29%)	2656 (25%)	10,696
	Essential worker parents	2672 (36%)	1194 (43%)	3866 (38%)	10,266
Outcomes	RCADS depression caseness	914 (13%)	447 (17%)	1361 (14%)	9786
	RCADS anxiety caseness	623 (9%)	334 (13%)	957 (10%)	9786
	Self‐reported worse wellbeing	2713 (36%)	1188 (42%)	3901 (38%)	10,415

*Note:* The term “*n*” refers to the total number included in each reported comparison between pupils at home versus those accessing in‐school provision; percentages reported are of the pupils who were in the relevant group (columns: home/school) and provided data for the relevant question (rows: factors).

Abbreviation: RCADS, Revised Children's Anxiety and Depression Scales.

### Educational provision

Those at home were compared with those in‐school (unadjusted frequencies) to assess the extent to which the group in‐school scored higher on the individual risk and contextual indicators (Table 1). 2,908 (27%) of the sample reported that they had left their homes to go to school, of which most were in years 10 (1136 = 39%) and 12 (568 = 20%). Of those receiving educational provision at home, 17% were in year 10 and 12% were in year 12. Most of the risk and contextual indicators were higher for those who had accessed in‐school provision. Compared with pupils at home full‐time, a slightly higher proportion of those in‐school reported ever experiencing food poverty (11% vs. 9%, *p* = .0001), not living with both parents (23% vs. 19%, *p* < .0001), receiving previous mental health support (29% vs. 23%, *p* < .0001) and parents being essential workers (43% vs. 36%, *p* < .0001). The prevalence of above the clinical threshold depression in this sample was greater for the in‐school group (17% vs. 13%, *p* < .0001), as was anxiety (13% vs. 9%, *p* < .0001). Pupils with in‐school educational provision were also more likely to report that their wellbeing was worse during lockdown (42% vs. 36%, *p* < .0001).

### Risk factors for mental health difficulties

Depression and anxiety, and self‐reported deterioration to wellbeing, were each modelled against the demographic and situational indicators. Adjusted ORs and 95% confidence intervals were calculated (see Table 2), where all indicators were accounted for together in each model.

**TABLE 2:** Logistic regression of mental health and self‐reported worse wellbeing with adjusted odds ratios for all risk and contextual factors

Outcome	Predictor	Adjusted odds ratio	95% Confidence interval	*p*‐Value
Depression (RCADS depression subscale *t*‐score ≥ 70) (*n* = 8798)	In‐school provision	1.16	1.00, 1.35	.0640
	Upcoming exams (year 10 or 12)	1.46	1.26, 1.69	<.0001
	Not living with both parents	1.20	1.03, 1.40	.0294
	Free school meals	1.03	0.81, 1.32	.7820
	Ever experienced food poverty	3.36	2.79, 4.04	<.0001
	Past access to mental health support	3.91	3.42, 4.47	<.0001
	Female gender	3.62	3.04, 4.32	<.0001
	Essential worker parents	1.34	1.18, 1.54	<.0001
Anxiety (RCADS anxiety subscale *t*‐score ≥ 70) (*n* = 8798)	In‐school provision	1.20	1.01, 1.42	.0495
	Upcoming exams (year 10 or 12)	1.49	1.24, 1.77	<.0001
	Not living with both parents	0.98	0.82, 1.17	.8151
	Free school meals	1.04	0.79, 1.37	.8151
	Ever experienced food poverty	3.03	2.48, 3.70	<.0001
	Past access to mental health support	3.83	3.29, 4.47	<.0001
	Female gender	2.55	2.10, 3.10	<.0001
	Essential worker parents	1.25	1.07, 1.46	.0063
Wellbeing got worse (*n* = 9309)	In‐school provision	1.10	0.98, 1.22	.0977
	Upcoming exams (year 10 or 12)	1.54	1.37, 1.73	<.0001
	Not living with both parents	1.31	1.17, 1.47	<.0001
	Free school meals	0.84	0.70, 1.00	.0515
	Ever experienced food poverty	1.82	1.57, 2.11	<.0001
	Past access to mental health support	1.77	1.60, 1.96	<.0001
	Female gender	1.88	1.70, 2.08	<.0001
	Essential worker parents	1.25	1.14, 1.37	<.0001

Abbreviation: RCADS, Revised Children's Anxiety and Depression Scales.

#### Depression and anxiety

Risk of depression was found to be higher for females (adjusted OR = 3.62, *p* < .0001), pupils who had previously accessed mental health support (adjusted OR = 3.91, *p* < .0001) and those who had ever experienced food poverty (adjusted OR = 3.36, *p* < .0001). Increased risk of depression was also found for pupils whose parents were likely essential workers (adjusted OR = 1.34, *p* < .0001), pupils who were approaching national examinations (adjusted OR = 1.46, *p* < .0001) and a small increased risk for pupils not living with both parents (adjusted OR = 1.20, *p* = .0294) that was not significant in the sensitivity analysis that included non‐response weights (OR = 1.18, *p* = .0565).

**FIGURE 1:**
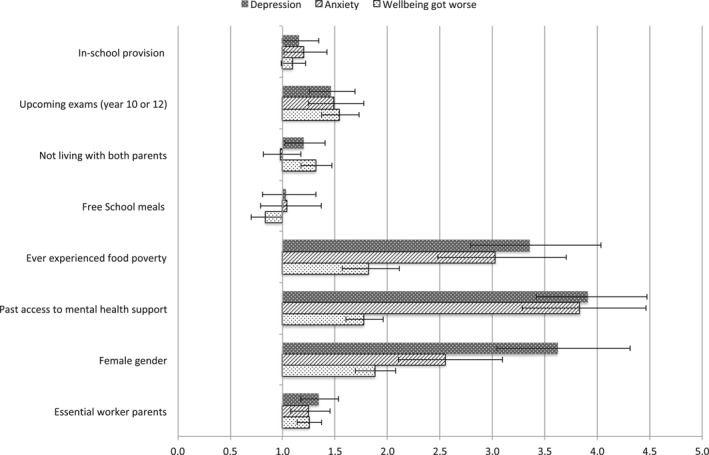
Adjusted odds ratios and 95% confidence intervals for depression, anxiety and self‐reported worse wellbeing during school closures

There was no association between self‐reported eligibility for free school meals and depression. The group allocated to in‐school educational provision did not show clear evidence of elevated risk of depression in this adjusted model (adjusted OR = 1.16, *p* = .0640). For the model predicting anxiety, the risk factors demonstrated a similar pattern to that seen for depression (Figure 1). Pupils not living with both parents were not at elevated risk of anxiety. Pupils accessing in‐school provision had a small but significant increased risk of anxiety (adjusted OR = 1.20, *p* = .0495), but this was not significant in the weighted analysis (OR = 1.20, *p* = .0514).

#### Self‐reported deterioration to mental wellbeing

Factors associated with increased risk followed a similar pattern for self‐reported deterioration of mental wellbeing during lockdown to the pattern for depression and anxiety (Figure 1). For factors especially relevant to the school lockdown, namely having key worker parents or preparing for key examinations in 2021, ORs for reporting wellbeing to be worse during lockdown were similar in magnitude to the clinical outcomes. For the deprivation and vulnerability predictors, ORs were smaller than those for the validated clinical measures.

## DISCUSSION

This study provides insight into the mental health and wellbeing of different groups during the partial school closures of the first UK COVID‐19 school lockdown in 2020 and how this relates to which pupils took up in‐school places. The results show that those who were accessing on‐site school were more likely to have depression or anxiety and were more likely to report a deterioration in their wellbeing. However, the poorer outcomes for the group in‐school were accounted for by pre‐existing vulnerability (e.g. experiencing food poverty and previous access to mental health support), in line with the practice during the first lockdown of trying to prioritise in‐school places to the pupils who most needed them. Increased risk for poor mental health was not only associated with the established indicators of vulnerability and deprivation but also associated with contextual factors especially relevant during the lockdown measures – pupils approaching national examinations and parents performing essential roles.

Pupils who were accessing on‐site school were hypothesised to represent a higher risk group due to some of the criteria used to allocate school places (especially vulnerability) and schools' knowledge of which pupils met those criteria. As predicted, the group accessing on‐site educational provision had both higher scores on pre‐existing risk and vulnerability factors and poorer mental health outcomes than their peers at home. However, with adjustment for each of the individual indicators, effects of accessing in‐school provision on mental health outcomes were not robust and any differences between the groups should be interpreted with caution. Besides the national criteria for vulnerable children and children of essential workers, many schools used other information, at their discretion, to allocate places to the most vulnerable (Husband, D., personal communication, 7 September 2020), which might have included implicit or explicit knowledge of pupils' current mental health. Further studies could aim to clarify how different adaptations of in‐school provision might have impacted mental health either positively or negatively.

Factors associated with the highest odds of a deterioration of mental wellbeing, as well as depression and anxiety, were established risk factors, confirming predictions that more vulnerable young people would be at greater risk of mental health problems during the partial school closures (Armitage & Nellums, 2020; Van Lancker & Parolin, 2020). We found that pupils who had previously accessed mental health support had almost four times the odds of reaching clinical thresholds for depression and anxiety and were also more likely to report a further deterioration of their wellbeing. This is consistent with the finding by YoungMinds that 80% of the 2036 adolescents surveyed with pre‐existing mental health needs felt that their mental health had become worse during the pandemic (YoungMinds, 2020). This deterioration may be related to the reduced school provision during lockdown, missing many of the usual school rituals and structures, including interactions with peers and school staff, structured and unstructured daytime activities and systems of support (Courtney et al., 2020; Golberstein et al., 2020; Lee, 2020; YoungMinds, 2020). Notably, female pupils were especially at increased risk of depression, consistent with other studies (Altemus et al., 2014). Self‐reported food poverty was higher than self‐reported eligibility for free school meals, and pupils reporting food poverty had more than three times the odds of having depression or anxiety. In contrast, self‐reported access to free school meals was not reliably related to any of the mental health outcomes and did not differ according to educational provision, possibly due to pupils not being fully aware of which category they might fall into.

In addition to the established risk factors of vulnerability, deprivation and gender, a slightly increased risk was identified on all three mental health outcomes for factors especially relevant to lockdown. The first of these is the increased risk for pupils in school years 10 and 12, who were invited back to school from June to help them prepare for key examinations in 2021. These pupils were hypothesised to represent a group at increased risk during lockdown because they might experience longer term impacts of school closures on their educational and vocational outcomes, a hypothesis which is in line with responses to some smaller surveys suggesting that young people's mental health was impacted during lockdown by concerns about academic performance (NHS Digital, 2020; YoungMinds, 2020). These concerns might relate to reduced instruction time for key components of the curriculum, which could be more detrimental for pupils approaching national examinations. Pupils whose parents were leaving the house to go to work most days during lockdown were also more likely to report their wellbeing to be worse, potentially reflecting concerns about their parents or increased stress and reduced support at home (Clemens et al., 2020). The fact that pupils of essential workers were at increased risk even when controlling for other key characteristics, including in‐school provision, supports the allocation of school places to these pupils during lockdowns and has implications for targeting support.

### Limitations

This survey reports cross‐sectional data and so causal interpretations cannot be inferred. This limitation was mitigated to some extent by addressing two issues. Firstly, demographic and situational predictors that could not have been influenced by current mental health or wellbeing (such as food poverty) were selected, and secondly, lockdown‐specific outcome measures of pupils' perceived change to their wellbeing were included. ORs for risk factors on pupils' perceived change to their wellbeing (which was a single item subjective measure) were notably smaller than for the validated, composite measures of depression and anxiety using the RCADS, which are more reliable. However, depression and anxiety measured by the RCADS might reflect accumulated influences on mental health from before and during lockdown. Therefore, in order to form an impression of the extent to which mental wellbeing was impacted by school closures, both the validated RCADS outcomes and the subjective measure of change to wellbeing were considered together. This analysis is also restricted to background situational factors that were predicted to increase the negative impacts of school closures on mental health for some adolescents and did not try to assess factors that might have mediated effects on wellbeing over the course of lockdown, such as relationships at home or school, which are even more difficult to interpret in cross‐sectional data.

A number of considerations should be taken into account before generalising these findings to broader school populations. This study recruited only a selection of schools and FECs in Southern England and excluded special schools. A small proportion of the study sample was over 18, for whom the experience of lockdown might have been moderated by increased access to some resources. It is important to keep in mind that our findings represent the wellbeing of a broad sample of school pupils and older students at FECs. Furthermore, 63% of respondents were female and, in order to protect participants' identity, neither ethnicity nor special educational needs provision were asked. The analysis sample did not include many pupils who were at home during the lockdown period and less engaged with their remote educational provision or did not have good access to WiFi or equipment. The percentage of respondents who reported being eligible for free school meals is comparable to the 2019 demographic for the schools included in analyses, but lower than the England statistic for 2019 (14%). However, this study does mitigate some of the biases common to adolescent mental health research carried out during the pandemic (Pierce et al., 2020), reaching some of the more generalisable and vulnerable school‐aged populations in a sample large enough to detect the effects of these vulnerabilities. Also, we attempted to address the role of non‐response bias on the results using a preliminary sensitivity analysis that included weights based on a selection of demographics that could be matched to the ONS census data, which did not affect our main conclusions.

Further research is needed, utilising mixed methodology, so that the experience of pupils attending in‐school educational provision, as well as understanding the broader needs of the many stakeholders involved, is fully explored. Young people will be key in identifying many of the possible ways that some of the additional risks can be addressed. Additional studies might be able to examine how school closures impact different aspects of pupils' lives and how these relate to mental health.

## CONCLUSION

Our findings highlight how young people already at increased risk of mental health difficulties due to existing deprivation and vulnerabilities were more likely to perceive their mental wellbeing to have deteriorated during lockdown, amplifying existing inequalities. In addition to the predicted vulnerabilities, two new risk groups were identified as being affected by the pandemic: adolescents approaching key examinations in the next academic year and those whose parents were likely performing essential roles during lockdown. This study raises awareness of the needs of those children who are most likely eligible for in‐school places during periods of national lockdown. Compared to their counterparts who are accessing educational provision at home, those meeting the criteria for in‐school places will likely need good access to a range of educational and social activities to alleviate some of the additional pressures experienced during national lockdown periods as well as access to pastoral support.

## CONFLICT OF INTEREST STATEMENT

The authors have declared that they have no competing or potential conflicts of interest.

## ETHICAL STATEMENT

Ethical approval was granted by the University of Oxford Medical Sciences Division Research Ethics Committee (Reference R62366/RE0010 and RE0011). Participating schools sent a detailed information sheet to parents of school pupils under 16 years of age, with contact details for the research team and instructions on how to opt‐out by contacting the school. All pupils were asked to watch a 3‐min video about the survey content and how the responses are stored and used. Pupils provided active assent to participate in the research (for pupils under 16) or informed consent (for those over 16) before starting the survey. It was made clear to pupils both before and within the survey that the research was voluntary and that they could skip any questions that they did not want to answer.

## AUTHOR CONTRIBUTIONS

Karen Mansfield and Mina Fazel conceptualised the study, acquired funding, designed and carried out the investigation and coordinated the research planning and project administration. Karen Mansfield, Danielle Newby, Christoph Jindra and Galit Geulayov performed data curation. Karen Mansfield, Danielle Newby, Christoph Jindra, Nemanja Vaci and John Gallacher developed the methodology and formal analysis models. Danielle Newby developed the analysis code and ran the models, including validation using a sensitivity analysis, with support from Karen Mansfield and Galit Geulayov. Karen Mansfield, Emma Soneson, John Gallacher and Mina Fazel prepared the original draft of the manuscript and contributed to the general presentation. Galit Geulayov, Danielle Newby, Christoph Jindra and Nemanja Vaci critically reviewed and edited the draft. All authors checked and approved the final version of the manuscript.

## Supporting information

Supplementary Material

## Data Availability

The data that support the findings of this study are available on request from the corresponding author as an anonymised data extract in accordance with research governance procedures. The data are not publicly available due to privacy or ethical restrictions.
