# Supplementary material for: COVID‐19 partial school closures and mental health problems: A cross‐sectional survey of 11,000 adolescents to determine those most at risk
Source: JCPP Adv. 2021 Jul 20;1(2):e12021. doi: 10.1002/jcv2.12021 (PMC8420157; doi:10.1002/jcv2.12021)
Supplement: Supplementary file 1 — Supplementary Material [file JCV2-1-e12021-s001.docx]

**Table S1**. Question text and response options for the measures included in the study.

| **Variable** | **Question text** | **Response options** |
| --- | --- | --- |
| **Predictors** |  |  |
| Gender | Are you a boy or a girl? | girl - boy |
| Eligible for Free School Meals | Do you have (i.e. are you eligible for) free school lunches? | Yes No Don't know |
| Experience of food poverty | Some young people go to school or to bed hungry because there is not enough food at home. How often does this happen to you? | Not at all-Once or twice-Sometimes-Most days-Every day |
| Living situation - not living with both parents | Who do you live with? | I live with both of my parents in one house - I live with one of my parents - My parents are separated and I live in two homes - I live with other relatives or adoptive parents - I live with other people (foster carers, foster home, childrens home) - I live in more than one place - I live somewhere else - I would rather not say |
| Previous access to mental health support | Have you ever received any mental health support? | Yes No |
| Pupils accessing in-school provision during lockdown | During lockdown, have you left the house to go to school? | Not at all - Once or twice - Sometimes - Most days - Every day |
| Parents likely essential workers | During lockdown, have your parent(s)/carer(s) left the house to go to work? | Not at all - Once or twice - Sometimes - Most days - Every day |
| Upcoming national examinations | Please enter your Year Group: | 8 9 10 11 12 13 |
| Year group | Please enter your Year Group: | 8 9 10 11 12 13 |
| School ID | Please enter your Username: | Unique school ID |
| **Outcomes** |  |  |
| Above clinical threshold depression | Please let us know how you've been feeling most of the time..(sypmtoms of depression) | Never - Sometimes - Often - Always |
| Above clinical threshold anxiety | Please let us know how you've been feeling most of the time..(sypmtoms of anxiety) | Never - Sometimes - Often - Always |
| Perceived deterioration of mental wellbeing | During lockdown, how happy have you been feeling in general (your mental well-being)? | Much worse - Slightly worse - The same - Slightly better - Much better |

**Table S2**. Measures included in the study and associated coding.

| **Type of Measure** | **Variable** | **Scoring** |
| --- | --- | --- |
| **Predictors** |  |  |
| Demographic | Gender | Female versus male (reference group) |
| Deprivation indicator | Eligible for Free School Meals | Yes (reference = no, don't know, or no response) |
| Deprivation indicator | Experience of food poverty | Binarised to create 2 groups: once or twice or more often vs 'not at all' (reference group) |
| Vulnerability indicator | Living situation - not living with both parents | Multiple response options scored as 'not living with both parents'  (Reference = 'living with both parents') |
| Vulnerability indicator | Previous access to mental health support | Yes vs No or no response (reference group) |
| Hypothesised risk | Pupils accessing in-school provision during lockdown | Binarised to create 2 groups: 'not at all' vs the rest (reference group) |
| Hypothesised risk | Parents likely essential workers | Binarised to create 2 groups: 'most days' or 'every day vs the rest (reference group) |
| Hypothesised risk | Upcoming national examinations | Year groups binarised to 2 groups: 10 & 12 vs. rest (reference group = years 8, 9, 11, 13) |
| Control variable | Year group | Year group used as random intercept |
| Control variable | School ID | School ID used as random intercept (91 schools in analysis sample) |
| **Outcomes** |  |  |
| Depression | Above clinical threshold depression | T-score above clinical threshold (≥70) based on 11 RCADS depression items |
| Anxiety | Above clinical threshold anxiety | T-score above clinical threshold (≥70) based on 14 RCADS anxiety items |
| Change to wellbeing | Perceived deterioration of mental wellbeing during lockdown | Binarised to create 2 groups: 'much worse' or 'slightly worse' vs the rest (reference group) |
